# Constitutive Modelling of Polylactic Acid at Large Deformation Using Multiaxial Strains

**DOI:** 10.3390/polym13172967

**Published:** 2021-08-31

**Authors:** John Sweeney, Paul Spencer, Glen Thompson, David Barker, Phil Coates

**Affiliations:** IRC in Polymer Science and Technology, Mechanical and Energy Systems Engineering, Faculty of Engineering and Informatics, University of Bradford, Bradford BD7 1DP, UK; p.e.spencer@bradford.ac.uk (P.S.); g.p.thompson@bradford.ac.uk (G.T.); d.barker2@bradford.ac.uk (D.B.); p.d.coates@bradford.ac.uk (P.C.)

**Keywords:** PLA, multiaxial, modelling, Eyring

## Abstract

Sheet specimens of a PLLA-based polymer have been extended at a temperature near to the glass transition in both uniaxial and planar tension, with stress relaxation observed for some time after reaching the final strain. Both axial and transverse stresses were recorded in the planar experiments. In all cases during loading, yielding at small strain was followed by a drop in true stress and then strain hardening. This was followed by stress relaxation at constant strain, during which stress dropped to reach an effectively constant level. Stresses were modelled as steady state and transient components. Steady-state components were identified with the long-term stress in stress relaxation and associated with an elastic component of the model. Transient stresses were modelled using Eyring mechanisms. The greater part of the stress during strain hardening was associated with dissipative Eyring processes. The model was successful in predicting stresses in both uniaxial and planar extension over a limited range of strain rate.

## 1. Introduction

The polylactide family of polymers is currently of strong interest. It has a number of environmental advantages that include its carbon-neutral plant-based origins and potential for both recycling and end-of-life composting [1,2,3]. These make it suitable for many consumer and packaging applications. It has a low glass transition temperature, making it convenient and popular for Fused Filament Fabrication [4]. Additionally, its bioresorbability has been exploited in medical devices for many years [5,6,7,8].

A good understanding of its mechanical behaviour is essential for design of load-bearing components and for the understanding of forming processes. In this paper we focus on temperatures near glass transition and large strains, corresponding to conditions relevant for solid phase processes such as thermoforming, blow-moulding or die-drawing. We shall report experiments conducted to large strains, with accompanying molecular orientation, strain hardening and the development of anisotropy.

For a constitutive model to be generally applicable, it must be fully three-dimensional. When deformations are large, mechanical behaviour may be complex and need to be modelled using multiple material parameters. The values of these parameters must be defined using experimental measurements. Uniaxial mechanical testing is most frequently used. However, such experiments impose only one specific form of strain field from the infinity of possibilities, which furthermore possesses a high level of symmetry. Despite this, uniaxial testing is frequently the sole source of experimental data when modelling general three-dimensional deformations. This has been shown to be unsatisfactory. Two different material parameter sets may generate stress predictions that are very similar for uniaxial strains, but very different for planar extension [9]. In the latter experiments, strain is held at zero in one of the directions normal to the stretching axis, so that all three principal strains are different, to give a strain field more general than for uniaxial strain and thus a more severe test of the material model.

In this paper, we evaluate a material model using both uniaxial and planar extension. In the latter experiments a biaxial testing machine is used to record forces both axially and transversely. In all cases stress relaxation is monitored at the final strain and the stresses allowed to decay with time to effectively constant levels. The various experimental results lead to the building and definition of a model consisting of dissipative and elastic components compatible with the observations.

For amorphous polymers above the glass transition, entropically-based theories of rubber elasticity have been used as components in material models that give realistic predictions of stress-strain relations [10,11]. In particular, the rubber elastic mechanism is effective in incorporating strain hardening effects into the models so that an accurate stress-strain response in loading is predicted. In these models, it is assumed that the stress associated with the strain–hardening mechanism is entropic and elastic. This assumption has been brought into question. Its validity has been investigated using simulations of amorphous polymer at the molecular level, which showed entropic stresses to be far lower than those associated with strain hardening [12,13]. This together with more extensive experimental findings strongly suggest that the predictions of strain hardening using entropic models are essentially fortuitous, a conclusion supported further by the simulation work of Mahajan and Basu [14]. It is also relevant here that an experimentally successful constitutive model has been developed of polymethyl methacrylate (PMMA) for which strain hardening has both viscous and entropic components, with the viscous component dominant [15].

To investigate dissipative effects experimentally, non-monotonic loading is most effective. Thus, Billon used tensile testing in which loading was followed by unloading to study PMMA near the glass transition [16], to assess a model in which the Edwards-Vilgis rubber elastic theory [17] was used as a framework and dissipative effects introduced into it. Alternatively, parameter values for viscoplastic models can be derived with the aid of stress relaxation [18] or stress dip experiments [19,20]. In this paper, stress relaxation is used to distinguish between elastic and dissipative processes.

Strain hardening in polymers is accompanied by molecular orientation and thence anisotropy. A material model that predicts strain hardening should also predict anisotropy, as does the model presented in this work. We use a biaxial testing machine to conduct large strain tensile experiments that measure both the anisotropic response and strain hardening, to evaluate these important aspects of the model predictions.

An unusual feature of this programme is that it combines biaxial tensile experiments and stress relaxation. The benefits of these techniques can be summarised as:

1. Biaxial testing, in the form of planar extension, provides a strain field that is more general than uniaxial testing and explores the effectiveness of the material model in more general conditions. The results provide a measure of anisotropy.
2. Stress relaxation allows for direct observation of the elastic component of the stress in the form of steady-state behaviour. This aids the derivation of material parameters and puts a limit on the extent of the entropic contribution to strain-hardening.

## 2. Materials and Methods

### 2.1. Material and Preparation

The polylactic acid was manufactured by NatureWorks (Minnetonka, MN, USA) as grade 4032D. It has been the subject of a number of studies of its mechanical properties (e.g., Wei et al. [21], Al-Itry et al. [22]). It has a 1.4 mol% D-lactide content and molecular masses ${\bar{M}}_{n}$ = 107,300 and ${\bar{M}}_{w}$= 183,180 [23].

The material was supplied in the form of pellets. Before processing, granules were dried overnight at 60 °C in a vacuum oven (Medline Scientific Ltd., Chalgrove, Oxon, UK). Sheets of nominal thickness of 0.5 mm were made by compression moulding using a 20 tonne hydraulic press (Moore, Birmingham, UK). The press platens were raised to a temperature of 200 °C, higher than the polymer melting point, before the granules, held between copper sheets, were placed between them. The press was then closed up to make thermal contact and held without pressure for 2 min. Pressure was then increased to 3.1 MPa over a 45s period and then held for 15 s. Sheets were cooled rapidly to room temperature using water at 12 °C and 0.1 MPa pressure plumbed into the platens.

### 2.2. Differential Scanning Calorimetry (DSC)

To investigate the glass transition and crystallinity of the sheet material, DSC was used (DSC Q20, TA Instruments, Elstree, Herts, UK). Specimens were heated from ambient 22 °C to 190 °C at 10 °C/min. The glass transition temperature T_g_ was measured as 62.8 °C. Some cold crystallisation was observed at 112.9 °C and the melt temperature was 167.9 °C. The associated enthalpies were used together with a value of 93.7 J/g for the PLLA crystal to calculate a crystallinity of 19.4% [24].

### 2.3. Uniaxial Experiments

Plane dogbone specimens of gauge length 80 mm and width 10 mm conforming to ISO527-2-1A were cut from the sheets using a dumbbell cutter. Uniaxial tensile tests were carried out within a fan oven at 60 °C using an Instron 5564 testing machine (Instron, High Wycombe, Bucks, UK). Temperature was sensed using K-type thermocouples and control was provided by a PID temperature controller (RS CAL 9900, RS Components, Corby, UK). For each experiment steady-state temperature conditions were obtained and the specimen was held in these conditions for 5 min prior to testing to ensure heating throughout its mass. A thermocouple independent from the controller was situated 1–2 mm from the specimen surface giving a digital readout (RS 206-3738 RS Components). In each case extension ratios of 2 were imposed at constant linear speeds. After stopping the crosshead the extension ratio was maintained and stress relaxation observed for a period of at least 600 s. The set of testing speeds used correspond to initial linear strain rates between 2.3 × 10^−^^3^ and 5.8 × 10^−2^ s^−1^. All tests were conducted at 60 °C.

### 2.4. Biaxial Experiments

**Figure 1:**
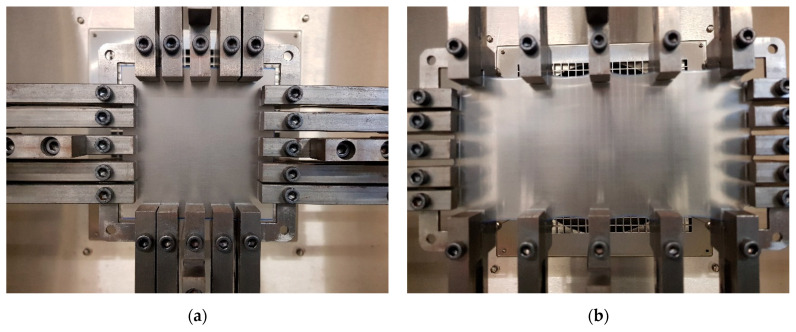
Specimen in biaxial machine (**a**) initially and (**b**) after planar stretch along the axial (horizontal) direction.

The biaxial stretching machine was designed and manufactured in-house. An initially square specimen is stretched along two perpendicular axes. For each axis, the specimen is pulled symmetrically on opposite sides using a pair of opposing load screws which drive loading rods connected to the stretching mechanism that holds the specimen. Each screw is driven by a Kollmorgen AKM21E servo motor controlled by a Kollmorgen AKD servo drive, the system being supplied by NI (Austin, TX, USA). The two axes are independently programmable with the potential for a wide range of displacement histories. The specimen is held by five pneumatic grips on each of its sides (see Figure 1) which are actuated by bottled nitrogen. The extension occurs within a large (1 m × 1 m× 0.6 m) fan oven (Harvard LTE, LTE Scientific, Oldham, UK) which can maintain steady temperatures indefinitely. An integral digital temperature controller senses temperature using a 100 Ω platinum resistive thermometer. Temperature is measured independently using a K-type thermocouple mounted at 1–2 mm from the centre of the specimen surface driving a digital display unit (RS 206-3738 RS Components). For each experiment steady-state temperature conditions were obtained and the specimen was held in these conditions for 5 min before the start of the test to ensure heating throughout the specimen mass. The loading rods are each instrumented with load cells outside the oven, with each cell sensing the total load on one specimen side. A program written in Labview (Labview 17.01f3, NI, Austin, TX, USA) runs on a PC. This controls the machine motion via a real time controller (CompactRIO, NI, Austin, TX, USA) using data supplied by the PC and captures the load data using a field-programmable gate array operating within the CompactRIO.

We conducted planar extension (constant width tension) experiments at 60 °C, stretching along either the horizontal or vertical axis and maintaining the transverse axis at zero strain, while monitoring the forces along both axes. Stretching was to a maximum extension ratio of 2, after which the axes were maintained stationary while the forces were monitored for 600 s as they relaxed. The specimens were of side 104 mm. Initial and final states of strain are shown in Figure 1. Stretching was at constant speed, corresponding to initial linear strain rates between 2.0 × 10^−3^ and 5.0 × 10^−2^ s^−1^, corresponding to the same range of octahedral strain rate as in the uniaxial experiments.

## 3. Results

### 3.1. Uniaxial Yield and Stress Relaxation

**Figure 2:**
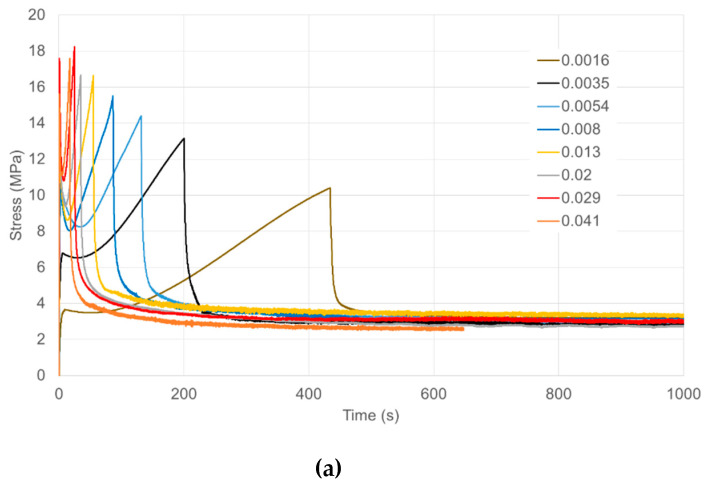

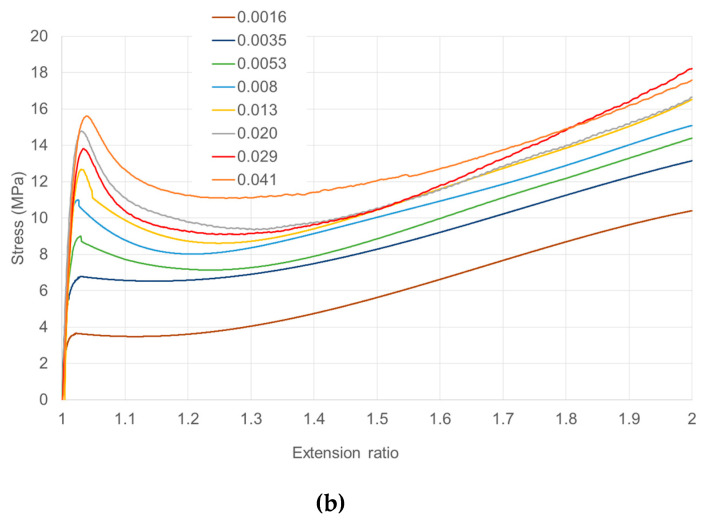
(**a**) Stresses in loading at a range of loading strain rates followed by stress relaxation at constant extension ratio 2. (**b**) Stresses in loading plotted against extension ratio. Octahedral strain rates (Equation (4)) are specified in s^−1^ in the captions. Times of loading range from 17.2 s at 0.041 s^−1^ to 442 s at 0.0016 s^−1^.

True stresses are plotted in Figure 2. Complete stress relaxation experiments are plotted against time in Figure 2a showing stresses during loading and then at constant strain, with the stress falling rapidly as the strain becomes constant. Stresses decay to essentially the same constant level after 600 s relaxation. Loading curves are plotted as stress against strain in Figure 2b.

Here yield stresses are easily discernible at small strains (~4–7%). Strain hardening is observed as the specimens extend. The yield stresses are strongly strain rate-dependent. At the higher strain rates, there is a drop in stress after yielding before strain hardening becomes dominant. Given the closeness of the testing temperature to the material’s glass transition, we consider this post-yield softening to be due to structural changes, as has been associated with a fictive temperature [25,26].

For consistent measures of strain rate in different stretching modes, we use the scalar octahedral strain rate $\dot{e}$, defined in terms of the velocity gradient tensor **L** by:

(1) $$\dot{e}=\sqrt{\frac{1}{3}L:L}$$

where **L** itself is defined in terms of the deformation gradient **F** as [27]

(2) $$L=\dot{F}F^{-1}$$

where the superposed dot denotes differentiation with respect to time. In principal directions I, II and III, (1) can be expressed as

(3) $$\dot{e}=\sqrt{\left({\dot{e}}_{I}^{2}+{\dot{e}}_{\mathrm{II}}^{2}+{\dot{e}}_{\mathrm{III}}^{2}\right)/3}$$

where ${\dot{e}}_{I},\;{\dot{e}}_{\mathrm{II}},\;{\dot{e}}_{\mathrm{III}}$ are true strain rates. For uniaxial incompressible stretching in the I direction:

(4) $$\dot{e}={\dot{e}}_{I}/\sqrt{2}$$

where ${\dot{e}}_{I},$ is the applied uniaxial strain rate. In these experiments, the testing speeds are constant and so the true strain rates vary. We characterise the experiments using the initial true strain rate, related to the extension ratio *λ*_I_ by ${\dot{e}}_{I}={\dot{\lambda }}_{I}$. We are also making the approximation of incompressibility. Scalar strain rates defined in this way give good comparisons at small strains and so are valid for analysing the yield behaviour.

### 3.2. Yield and Stress Relaxation in Planar Extension

In these experiments stretching is along the 1 axis at a strain rate ${\dot{e}}_{1},$ while the extension along the transverse 2 axis is held at ${\dot{e}}_{2}=0$ Under these conditions, for incompressible flow Equation (3) becomes:

(5) $$\dot{e}={\dot{e}}_{1}\sqrt{\frac{2}{3}}$$

**Figure 3:**
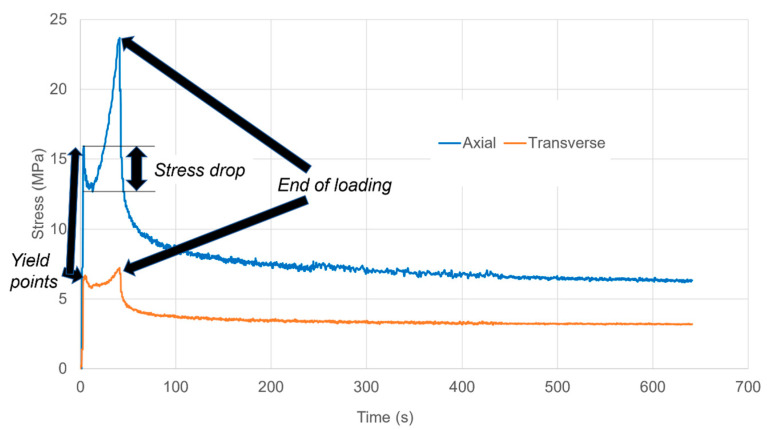
Stress relaxation in planar extension after straining at an octahedral strain rate of 0.010 s^−1^, showing yield points, subsequent drop in stress followed by strain hardening and stress relaxation.

A typical result is shown in Figure 3 for the linear strain rate ${\dot{e}}_{I}=0.013\;s^{-1}$ corresponding to $\dot{e}=0.010\;s^{-1}$. The yield, stress drop (strain softening) and strain hardening phenomena are present as with uniaxial stretching.

**Figure 4:**
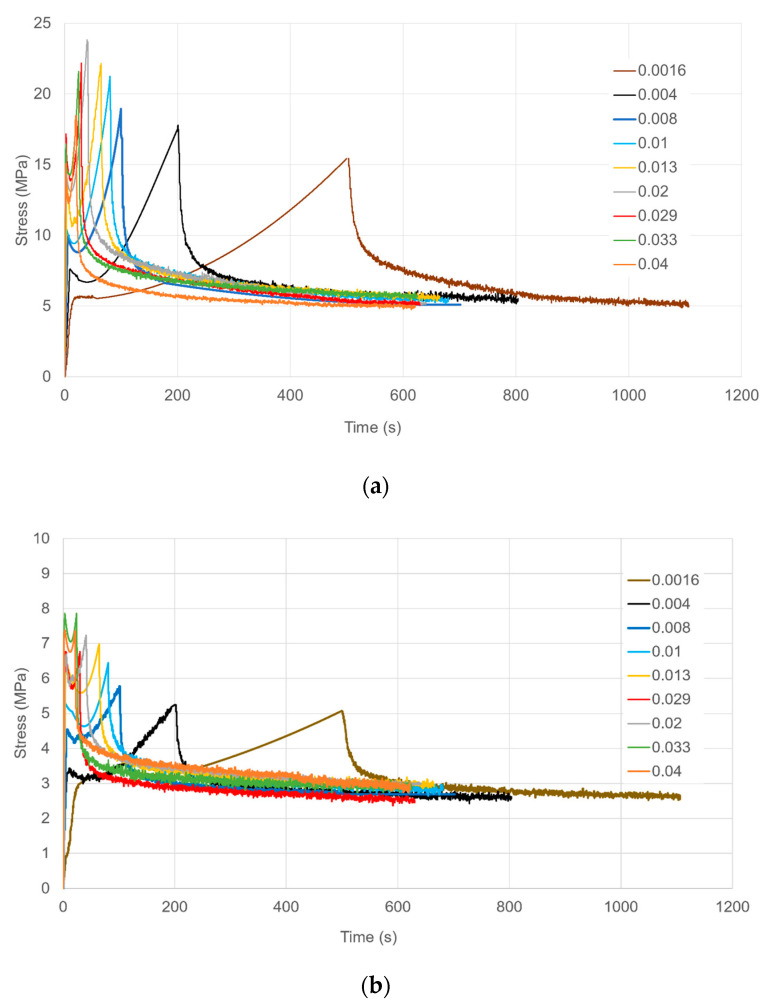
Axial (**a**) and transverse (**b**) true stresses for loading and stress relaxation in planar extension. They key shows octahedral strain rates in s^−1^.

**Figure 5:**
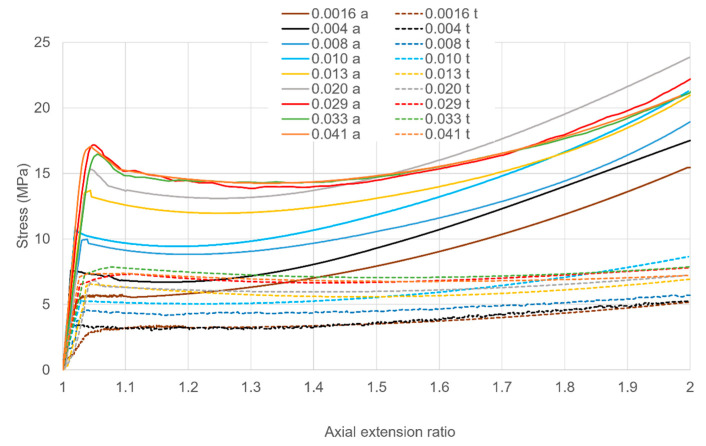
Stress-strain curves during loading for axial (full line, a) and transverse (broken line, t) stresses. They key shows octahedral strain rates in s^−1^. Times of loading range from 19.9 s at 0.041 s^−1^ to 510 s at 0.0016 s^−1^.

**Figure 6:**
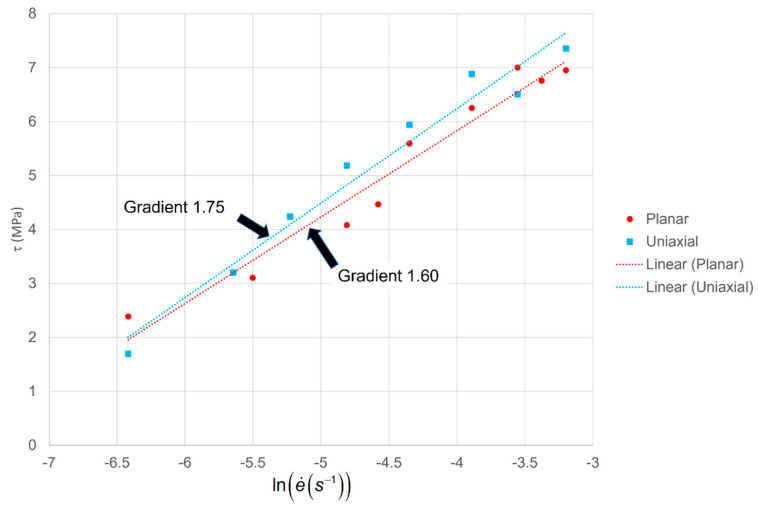
Plots of octahedral shear stress at yield against logarithm of octahedral shear strain rate for planar and uniaxial stretching.

Figure 4a,b respectively. Stress-strain behaviour during loading is shown in Figure 5. Yield stresses are readily identified and increase consistently with strain rate. Post-yield stress drops are observed at the higher strain rates as with the uniaxial results of Figure 2b. In Figure 5, stresses at the maximum strain increase with strain rate for rates up to 0.02 s^−1^, and then drop at higher rates. We attribute this effect to adiabatic temperature rise; this is a well-known source of nonlinearity in strain rate dependence in yield and drawing stress [28]. There is a smaller level of irregularity in rate dependence at the highest strain for the yield stress, visible in Figure 6; this is consistent with the small strain on yield and the consequent small level of strain energy available for conversion to heat. The effect is also absent in the uniaxial results of Figure 2b; this is consistent with different air flow rates in the ovens of the Instron and the biaxial machine, resulting in different levels of heat transfer between the specimens and the surrounding air.

## 4. Analysis of Yield

Here we analyse yield for both stretching modes in terms of the octahedral shear stress *τ* defined in terms of the stress deviator tensor **S** as:

(6) $$\tau =\sqrt{\frac{1}{3}S:S}$$

or for principal stresses *σ*_I_, *σ*_II_ and *σ*_III_ [28]:

(7) $$\tau =\frac{1}{3}\sqrt{{\left(\sigma_{I}-\sigma_{\mathrm{II}}\right)}^{2}+{\left(\sigma_{\mathrm{II}}-\sigma_{\mathrm{III}}\right)}^{2}+{\left(\sigma_{\mathrm{III}}-\sigma_{I}\right)}^{2}}$$

For uniaxial stretching along I this is equivalent to:

(8) $$\tau =\frac{\sqrt{2}}{3}\sigma_{I}$$

In planar extension in the I-II plane *σ*_III_ = 0 and Equation (7) gives:

(9) $$\tau =\frac{\sqrt{2}}{3}\sqrt{\sigma_{I}^{2}+\sigma_{\mathrm{II}}^{2}-\sigma_{I}\sigma_{\mathrm{II}}}$$

Yielding occurs at small strain (<0.07) and we may assume that the material is essentially isotropic at this stage. A conventional isotropic flow rule, such as Levy-Mises, then applies to give a value for transverse stress half that of the axial stress. For stretching along I:

(10) $$\sigma_{\mathrm{II}}=\sigma_{I}/2$$

and the expression (8) becomes:

(11) $$\tau =\frac{\sigma_{I}}{\sqrt{6}}$$

to give an alternative expression to (9) with little error at small strain.

In Figure 6, octahedral shear stresses at yield are plotted against scalar strain rates as defined in Equations (4) and (5) for both stretching modes. We analyse them using an Eyring process with pressure and shear activation volumes *v_p_* and *v_s_*, respectively that are related to model parameters *V_s_*, *V_p_* by vs. = *v_s_*/*kT* and *V_p_* = *v_p_*/*kT*. The octahedral strain rate and octahedral shear stress are related by [28]:

(12) $$\dot{e}={\dot{e}}_{0}\exp\left(V_{p}\bar{\sigma }\right)\sinh\left(V_{s}\tau \right)$$

where $\bar{\sigma }$ is the hydrostatic stress component and ${\dot{e}}_{0}$ is a term combining temperature and the free enthalpy barrier. For large arguments the hyperbolic sine function can be replaced by an exponential to give:

(13) $$V_{p}\bar{\sigma }+V_{s}\tau =\ln\left(2\dot{e}/{\dot{e}}_{0}\right)$$

Under uniaxial conditions, $\bar{\sigma }=\sigma_{I}/3$ and Equation (8) leads to

(14) $$\tau =\frac{\ln\left(2\dot{e}/{\dot{e}}_{0}\right)}{V_{p}/\sqrt{2}+V_{s}}$$

In planar extension, applying Equations (10) and (11) in Equation (13) gives:

(15) $$\tau =\frac{\ln\left(2\dot{e}/{\dot{e}}_{0}\right)}{V_{p}\sqrt{3/2}+V_{s}}$$

According to this implementation of the Eyring process, the gradients of the two Eyring plots in Figure 6 should have different gradients corresponding to Equations (14) and (15), giving a potential method for identifying separate values of *V_p_* and *V_s_*. However, given that *V_p_* is generally small compared with *V_s_*, the difference in gradients can be expected to be small.

Linear least squares fits for the two data sets give gradients as shown in Figure 6. The gradient of the planar extension results is less than that of the uniaxial ones, as expected according to Equations (14) and (15). Analysis using these equations gives a ratio *V_p_*/*V_s_* = 0.21. This should be viewed as the most likely value rather than a definitive result since, according to statistical analysis using the standard errors of the slopes [29], the gradients are not significantly different at the 10% level.

The ratio β = *V_p_*/*V_s_* has been evaluated by various workers for a range of polymers. Nazarenko et al. [30] used high pressure measurements on glassy polycarbonate to arrive at a ratio of 0.06. Also working with high pressure measurements, Truss et al. [31] derived β ratios of 0.035 and 0.063 for two Eyring processes in a model of polyethylene. Bauwens-Crowet et al. [32] used tensile and compressive experiments to arrive at the value 0.075 for polycarbonate, while noting the range 0.05–0.072 obtained by other workers. Buckley et al. obtained a ratio of 0.19 for PET [33] and Ho et al. a value of 0.10 for PS [34].

Against this background, the ratio value 0.21 is feasible. vs. and *V_p_* are then calculated from Equations (14) and (15) as 0.50 and 0.10 MPa^−1^ respectively. The value of vs. corresponds to an activation volume of 2.3 nm^3^. This is comparable with the values for PLLA of 1.75 and 4.75 nm^3^ in the multiprocess model of van Breeman et al. [35].

## 5. Modelling

### 5.1. Elementary Formulation

Polymers are mechanically time-dependent, showing creep and stress relaxation effects. They can be treated as viscoelastic, provided nonlinear theories are used for any appreciable level of strain. Fractional calculus has been used to extend the linear theory (e.g., [36]) and also in the nonlinear case [37]. However, most polymers, including the subject of our study, show yield phenomena. While viscoelastic theory, including the fractional calculus approach, has been extended to these so-called viscoplastic materials [38], there are other avenues that lend themselves naturally to describe the kind of rate-dependent yielding often observed in polymers. Prime among these is the Eyring process [39], which generates predictions of yield stress that are linear functions of the logarithm of strain rate, in line with many studies of polymers (e.g., [33]). It also provides an effective model for stress relaxation curves [20,40].

**Figure 7:**
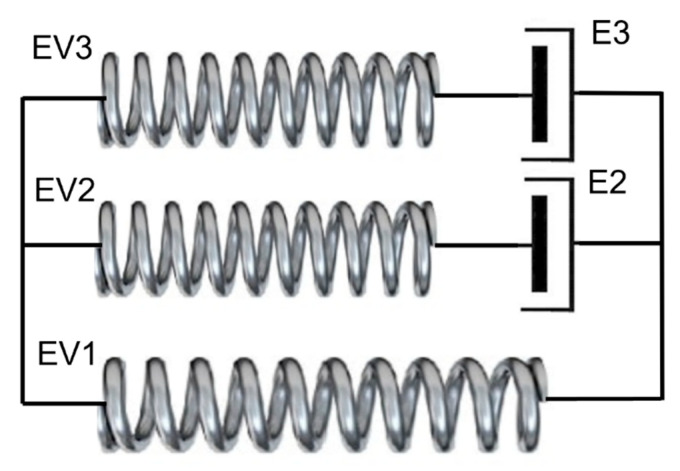
Three-arm model model with Arm 1 as an Edwards-Vilgis model and Arms 2 and 3 each as an Edwards-Vilgis in series with an Eyring process.

The results in Figure 2a, Figure 3 and Figure 4 show that the stress can be separated into an initial transient response and a longer term steady state response. We make use of this concept to construct the constitutive model by using the Eyring process for the transient response and representing the long term response using hyperelasticity. We propose the configuration expressed diagrammatically in Figure 7. It is subject to a deformation gradient F with principal values *λ*_I_, *λ*_II_, *λ*_III_.

The Edwards-Vilgis [17] model is used for the elastic processes. We apply a compressible form of the model, by separating the deformation gradient **F** into volumetric and deviatoric components. For principal extension ratios *λ*_I_, *λ*_II_, *λ*_III_ the volume ratio *J* is given by:

(16) $$J=\lambda_{I}\lambda_{\mathrm{II}}\lambda_{\mathrm{III}}$$

The deviatoric extension ratios ${\dot{\lambda }}_{I},\;{\dot{\lambda }}_{\mathrm{II}},\;{\dot{\lambda }}_{\mathrm{III}}$ are expressed as:

(17) $${\tilde{\lambda }}_{i}=\lambda_{i}J^{-1/3}(i=I,\;\mathrm{II},\;\mathrm{III})$$

which are the diagonal components of the incompressible deformation gradient $\tilde{F}$ so that *λ*_I_ · *λ*_II_ · *λ*_III_ = 1.

We now follow the method described by Kaliske and Rothert [41] to create a strain energy density function separated into deviatoric and volumetric components. For the Edwards-Vilgis model, the change U in strain energy density resulting from the deformation is then:

(18) $$U=\frac{N_{c}}{2\left(1-\alpha^{2}\sum_{i=I}^{\mathrm{III}}{\tilde{\lambda }}_{i}^{2}\right)}\sum_{i=I}^{\mathrm{III}}{\tilde{\lambda }}_{i}^{2}+\frac{N_{s}}{2\left(1-\alpha^{2}\sum_{i=I}^{\mathrm{III}}{\tilde{\lambda }}_{i}^{2}\right)}\sum_{i=I}^{\mathrm{III}}\left[\frac{\left(1+\eta \right){\tilde{\lambda }}_{i}^{2}}{1+\eta {\tilde{\lambda }}_{i}^{2}}+\ln\left(1+\eta {\tilde{\lambda }}_{i}^{2}\right)\right]+\frac{B}{2}{\left(\ln J\right)}^{2}$$

where *N_c_* and *N_s_* are proportional to crosslink and sliplink densities respectively, *α* is a parameter that controls finite strain extensibility, *η* defines the mobility of the sliplinks (with *η* = 0 corresponding to a fixed crosslink) and *B* is the bulk modulus. The energy density can be considered as the sum of a deviatoric component $\tilde{U}$ and a volumetric component:

(19) $$U=\tilde{U}+\frac{B}{2}{\left(\ln J\right)}^{2}$$

Principal stresses *σ*_I_, *σ*_II_ and *σ*_III_ are given by:

(20) $$\sigma_{i}=\frac{\lambda_{i}}{J}\frac{\partial U}{\partial \lambda_{i}}=\frac{\lambda_{i}}{J}\frac{\partial \tilde{U}}{\partial \lambda_{i}}+\frac{B\ln J}{J}(i=I,\;\mathrm{II},\;\mathrm{III})$$

and we may identify the deviatoric and mean components *s_i_* and $\bar{\sigma }$, respectively, as:

(21) $$s_{i}=\frac{\lambda_{i}}{J}\frac{\partial \tilde{U}}{\partial \lambda_{i}}(i=I,\;\mathrm{II},\;\mathrm{III})$$

(22) $$\bar{\sigma }=\frac{B\ln J}{J}$$

where:

(23) $$s_{i}=\sigma_{i}-\bar{\sigma }(i=I,\;\mathrm{II},\;\mathrm{III})$$

Equations (21) and (22) define the response of the hyperelastic elements. For the elements EV1 in Figure 7, volume change and hydrostatic stress are included and the full strain energy density expression (18) applies. For the other Edward-Vilgis processes, the strains are incompressible and the stresses purely deviatoric, so that the strain energy density is $\tilde{U}$.

For the Eyring elements, the stress and strain rate are related by Equation (12). The components of strain rate are specified by the Levy-Mises flow rule:

(24) $$\begin{array}{l} {\dot{e}}_{I}^{p}={\dot{e}}^{p}s_{I}/\tau \\ {\dot{e}}_{\mathrm{II}}^{p}={\dot{e}}^{p}s_{\mathrm{II}}/\tau \\ {\dot{e}}_{\mathrm{III}}^{p}={\dot{e}}^{p}s_{\mathrm{III}}/\tau \end{array}$$

For each model arm containing an Eyring and an Edwards-Vilgis process, equilibrium is imposed by equating the deviatoric stress components. The deformation is split into plastic and elastic parts so that for the 2 arm in Figure 7:

(25) $$\tilde{F}=F^{p2}F^{e2}$$

(26) $$\tilde{F}=F^{p3}F^{e3}$$

Expressions for the elastic stresses were derived by implementing Equation (20) using the symbolic algebra package Maple. A time-marching numerical scheme is used to implement the model. Plastic strain rate is derived from the differences in plastic strain between successive increments. Arms 2 and 3 are associated with stress deviator tensors **S^2^** and **S^3^** respectively. For arm 1, the stress tensor **Σ^1^** has principal components defined by Equation (20). The total stress **Σ** in the model is then:

(27) $$\Sigma =\Sigma^{1}+S^{2}+S^{3}$$

Plane stress conditions are assumed with zero total stress in the III direction. Iterative procedures are used to impose this condition and to produce equilibrium of the hyperelastic and plastic stress deviators in arms 2 and 3.

### 5.2. Results for Elementary Model

The parameters for the Edwards-Vilgis elastic arm EV1 are derived from the long-term steady-state stresses in both uniaxial and planar experiments. We first establish the bulk modulus *B* in Equation (18) from the initial elastic material response on fast loading, assuming a Poisson’s ratio of 0.45. We assume a sliplink-only model with *N_c_* = 0. The three stress values—planar axial, planar transverse and uniaxial—are then averaged to provide sufficient information to specify *N_s_*, α and η in Equation (18). Values are given in Table 1.

The 1 arm has the parameters of Table 1 corresponding to the long-term elastic response. The 2 arm has Eyring parameters vs. and *V_p_* corresponding to the analysis of yield of Section 4 above, and an ${\dot{e}}_{0}$ term (Equation (12)) a value such as to give a realistic level of yield stress. For the Edwards-Vilgis network in the 2 arm, *N_s_* = α = 0 to give a Gaussian model with *N_c_* value such as to give a realistic initial stiffness response in accordance with the experimental observations. The model parameters are specified in Table 1.

**Figure 8:**
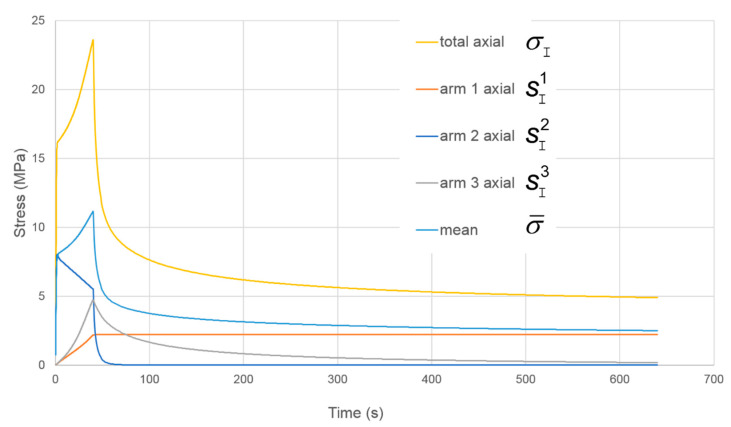
Stress components for the model of Figure 7.

The model is subject to planar extension to an axial extension ratio of 2 at a constant rate ${\dot{\lambda }}_{I}$ corresponding to an initial octahedral strain rate $\dot{e}$ = 0.021 s^−1^. Axial stresses are shown in Figure 8 in terms of deviatoric components from the three arms together with the mean stress $\bar{\sigma }$. Arm 2 produces the initial yield behaviour. Here yielding is followed by a fall in stress; the Eyring process is operating at its yield point, which falls as the true strain rate falls, in accordance with the constant applied ${\dot{\lambda }}_{I}$ and increasing *λ*_I_. Thus no strain hardening is available from this arm, and very little from arm 1, given its low value of stress. Arm 3 is introduced to account for the strain hardening and to give a more realistic rate of stress relaxation after loading. Its parameters in Table 1 are such as to give realistic axial and transverse stresses. The major part of the stress from strain hardening arises from arm 3, making strain hardening a largely dissipative process.

While this model is capable of giving useful predictions, its major failure is that it does not predict stress drops after the initial yield (see Figure 3). This feature will be addressed in the next section.

### 5.3. Modelling with Stress Drops

The post-yield stress drops such as we have observed have been associated with structural changes. The changed structural state of the material has been shown to be represented to a good approximation by a change of temperature to a ‘fictive temperature’ [42]. In constitutive modelling of polymers, the fictive temperature has been assumed to evolve as a function of the plastic strain [25,26]. In this model we do not directly involve fictive temperature, but rather allow activation volume to evolve as a function of plastic strain. In particular, $V_{s}^{2}$, the shear activation volume in arm 2, is assigned to be a function of the plastic strain in the Eyring process in arm 2. The appropriate measure of strain is the scalar octahedral plastic strain *e^p^* given by:

$$e^{p}=\frac{2}{3}\sqrt{{\left(e_{I}^{p}-e_{\mathrm{II}}^{p}\right)}^{2}+{\left(e_{\mathrm{II}}^{p}-e_{\mathrm{III}}^{p}\right)}^{2}+{\left(e_{\mathrm{III}}^{p}-e_{I}^{p}\right)}^{2}}$$

where $e_{I}^{p},e_{\mathrm{II}}^{p},e_{\mathrm{III}}^{p}$ are principal logarithmic plastic strains. For the 2 arm, with incompressible plastic flow, this becomes:

(28) $$e^{p2}=2\sqrt{\frac{2}{3}\left[{\left(e_{I}^{p2}\right)}^{2}+{\left(e_{\mathrm{II}}^{p2}\right)}^{2}+e_{I}^{p2}e_{\mathrm{II}}^{p2}\right]}$$

For the functional form of the evolution of the activation volume, we have adapted the approach used by Turner et al. [26] for the evolution of the fictive temperature. Then:

(29) $$V_{s}^{2}=V_{s0}+\Delta V_{s}\left(1-\exp\left[-{\left(\frac{e^{p2}}{e_{0}^{p}}\right)}^{r}\right]\right)$$

Here, the initial activation volume $V_{s_{0}}$ corresponds to the initial yield and is equal to the value of vs. given for arm 2 in Table 1. Adjustable parameters are: Δ*V_s_*, the maximum change in shear activation volume; $e_{0}^{p}$, which controls the strain range over which the change in activation volume occurs; and r, a fitting parameter of order unity. Using this approach, the stress drop is related to structural change via the plastic strain, but without explicit intermediation of the fictive temperature. The revised model is essentially that of the previous Section 5.2 with the addition of the variation in $V_{s}^{2}$ as defined by Equation (29). The parameter values of Table 1 apply and the values of the additional parameters are given in Table 2.

#### 5.3.1. Uniaxial Strains

**Figure 9:**
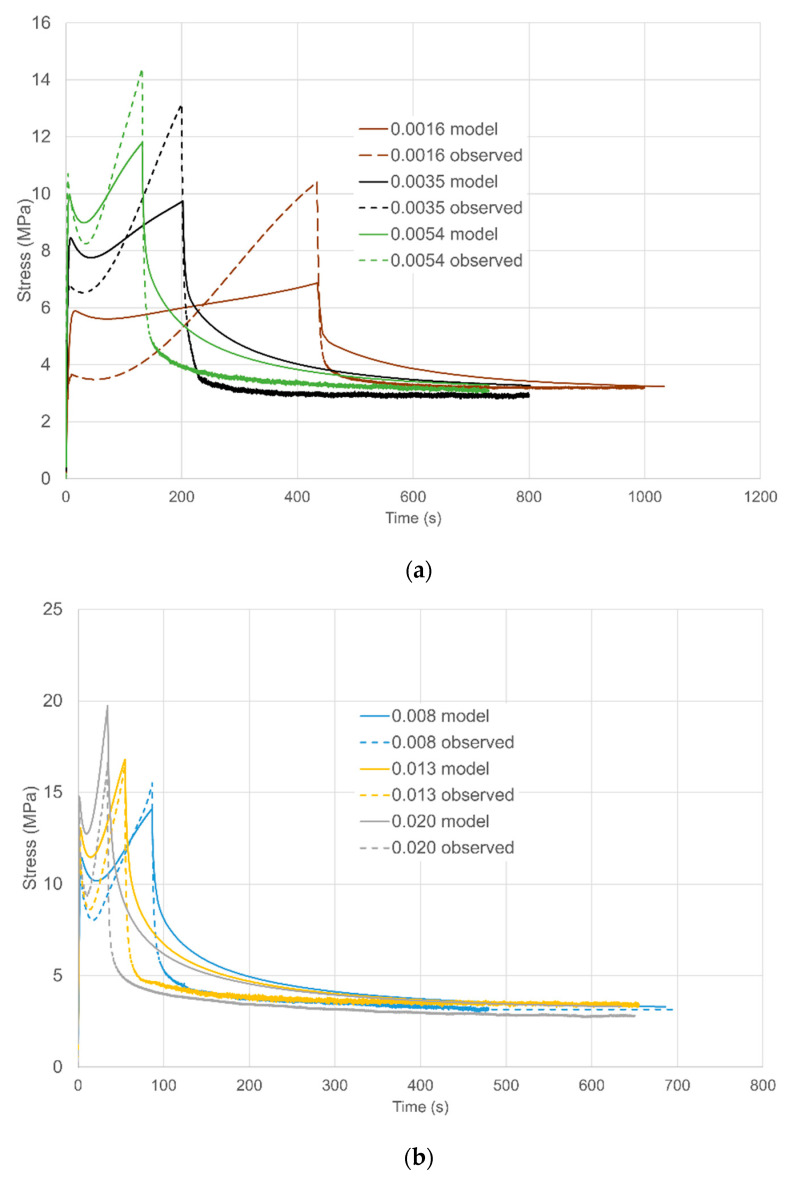

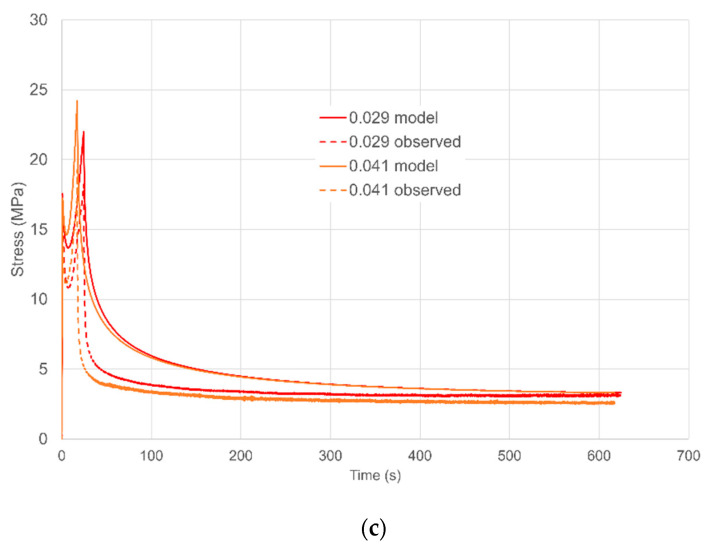
Uniaxial loading and stress relaxation. The key shows octahedral shear rates in s^−1^. (**a**) Three lowest strain rates; (**b**) three intermediate strain rates; (**c**) two highest strain rates.

For the three lowest strain rates, (1.6–5.4 × 10^−3^ s^−1^) observations and predictions are shown in Figure 9a as stress against time. The predicted initial yields are significantly low at the two lowest strain rates, corresponding to values of shear yield stress below the fitting line in Figure 6. More significantly, predicted levels of strain hardening are low. The level of stress drop increases with strain rate, in line with observations.

**Figure 10:**
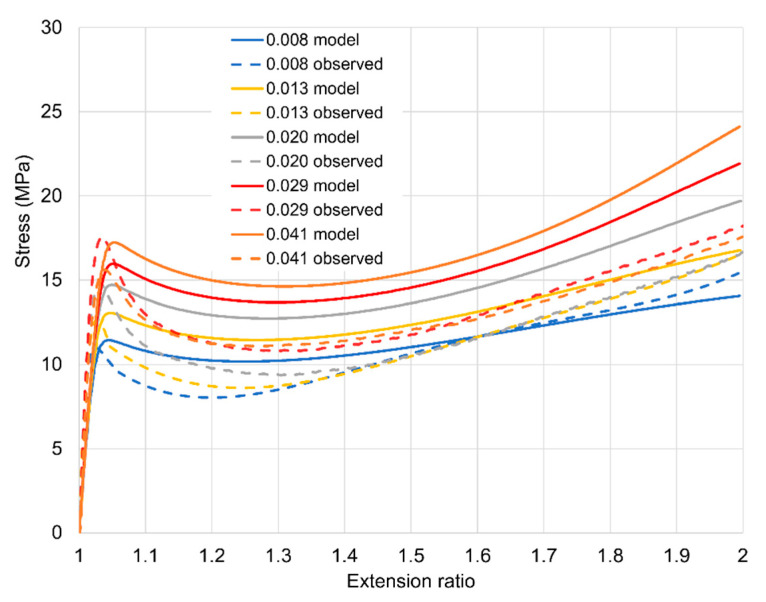
Uniaxial loading for the five highest strain rates, shown as octahedral rates in the key in s^−1^.

Figure 9b shows stress against time for the next three rates (8.0 × 10^−3^–2.0 × 10^−2^ s^−1^) while the loading curves are included in Figure 10 as stress against strain for clarity. Predictions are closer to observations at these rates. A major defect is the low predictions of stress drop; this is related to the value of Δ*V_s_*, which also controls the levels predicted for planar extensions. Similar comments apply to the highest rates (2.9 × 10^−2^ and 4.1 × 10^−2^ s^−1^) in Figure 9c and Figure 10.

#### 5.3.2. Planar Strains

For the three lowest strain rates, (1.6–8.2 × 10^−3^ s^−^^1^) observations and predictions are shown in Figure 9a as stress against time. The quality of the predictions is similar to the uniaxial results of Figure 9a in terms of the strain hardening, but the model levels of stress drop are more realistic. The quality of the modelling improves with increasing strain rate so that at 8.2 × 10^−3^ s^−1^ predictions are at a useful level.

**Figure 11:**
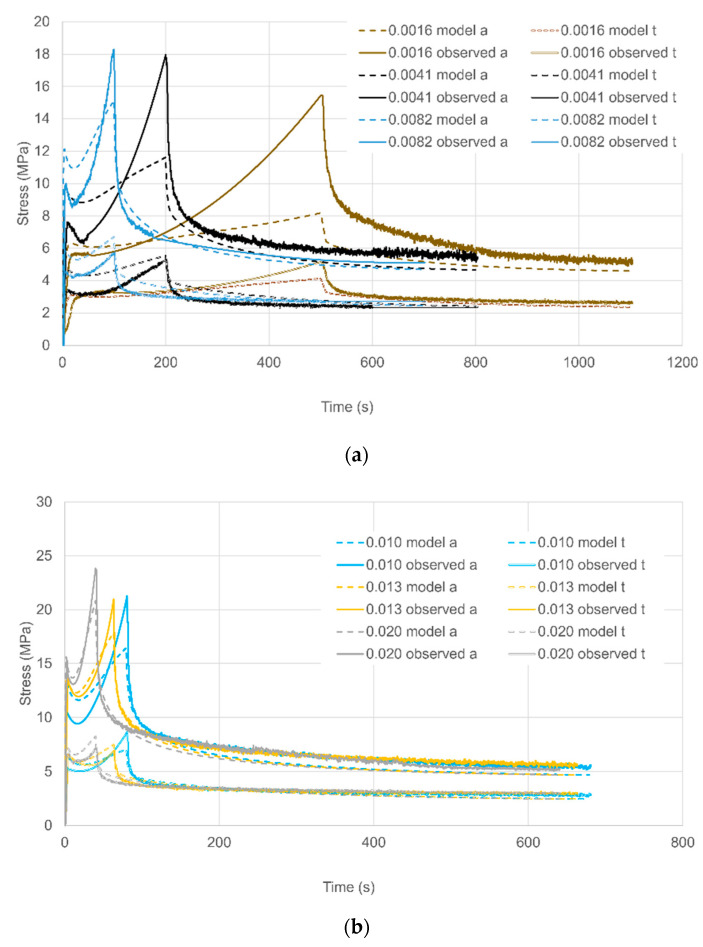

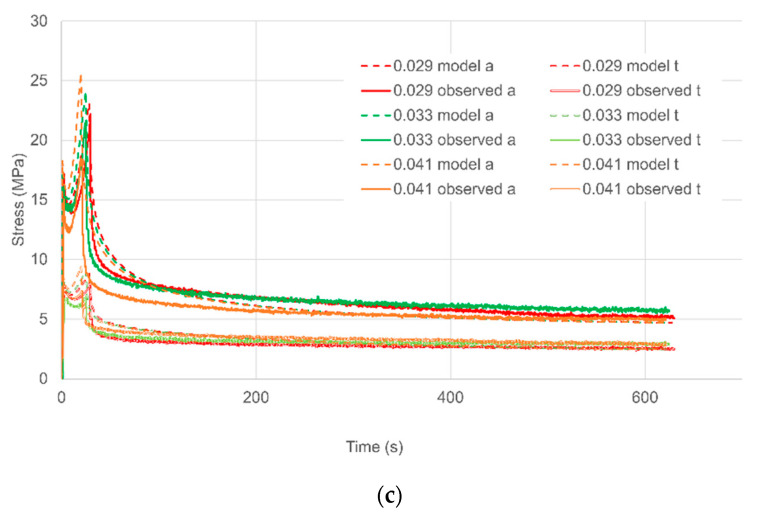
Planar loading and relaxation. The key shows octahedral strain rates in s^−1^ and also identifies axial and transverse stress (a and t respectively). (**a**) the three lowest, rates; (**b**) three intermediate rates and (**c**) three highest rates.

**Figure 12:**
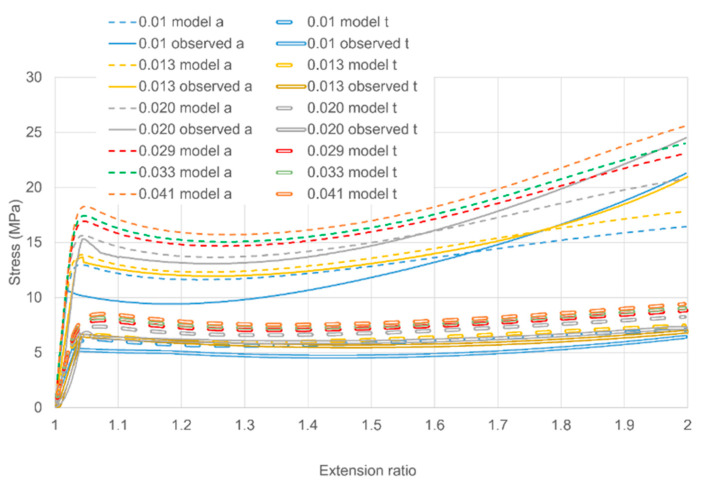
Planar stress-strain curves in loading for the six highest strain rates. The key shows octahedral strain rates in s^−1^ and also distinguishes axial (a) and transverse (t) stresses. The observations at the three highest rates are omitted as they are inconsistent with those at lower rates, apparently because of adiabatic heating.

Results for strain rates in the range 1.0–2.0 × 10^−2^ s^−1^ are presented in Figure 11b show an increased accuracy for the model. At the three highest rates (2.9–4.1 × 10^−2^ s^−1^) in Figure 11c the experimental stresses are not monotonic functions of strain rate, an effect that was discussed in Section 3.2 and attributed to adiabatic heating. This leads to high prediction s of stress at 3.3 and 4.1 × 10^−2^ s^−1^. Figure 12 shows stress-strain curves for the data in Figure 11b,c to show the loading behaviour. This figure shows clearly the low experimental stresses at the three highest rates. However, the magnitudes of the stress drops are reasonably well modelled.

A general feature of the results is that the model fits are closer at longer times. As steady state is approached, the elastic stress components become dominant and so the predictions are closer to the fitted long-term values. At shorter times, the material becomes more challenging from the point of view of modelling as the transient terms become important. This is particularly so during loading, as the stress drop phenomenon is an additional factor. As a result the modelling of the stress-strain behaviour of Figure 12 is generally less accurate than the stress relaxations of Figure 11.

## 6. Discussion and Conclusions

The model gives useful predictions at octahedral strain rates above 0.1 s^−1^. With the current set of parameters, the representation of planar extension experiments is more effective than that for uniaxial tests. It would be possible to derive a set of parameter values that gave a better representation of the uniaxial results, but only at the expense of the effectiveness for planar stretching. The obstacle to a consistently good performance over both stretching modes is that, in the uniaxial case, the stress drops observed experimentally are larger. This illustrates that the use of more than one stretching mode provides a more rigorous test of a material model. It also raises the question of whether the difference in size of the stress drops observed in different stretching modes is a feature of other polymers. A positive feature of the present approach to modelling of the stress drops is that it reproduces the increase in size of drop as the strain rate increases.

The use of stress relaxation enables the observed stress to be split into transient and steady-state components, with the latter represented by a single hyperelastic process. This limits the elastic component of the stress during straining, and forces the strain hardening process to be largely dissipative; it is represented by an arm of the model that contains an Eyring process. This approach is in line with the findings from simulations that strain hardening is not associated with entropy [12] and is dominated by viscoplasticity [13].

To effectively model solid phase processes at large strain, multiaxial deformations need to be understood quantitatively. Many processes, such as stretch blow-moulding, involve multiple steps and so give opportunities for significant stress relaxation, which as shown here can be very rapid. The simultaneous investigation of both multiaxial and stress relaxation behaviour has potential to provide highly relevant modelling data and verification.

## Figures and Tables

**Table 1:** Three-arm model parameters.

	*N_c_* MPa	*N_s_* MPa	B MPa	*α*	*η*	*V_s_* MPa^−1^	*V_p_* MPa^−1^	${\dot{e}}_{0}\;s^{-1}$
Arm 1	0	4.48	950	0.216	1.60	-	-	-
Arm 2	190	0		0	0	0.50	0.10	7 × 10^−4^
Arm 3	1.0	0.0		0.36	0.0	0.25	0.05	4 × 10^−3^

**Table 2:** Stress drop data.

$V_{s0}\;{\mathrm{MPa}}^{-1}$	$\Delta V_{s}\;{\mathrm{MPa}}^{-1}$	$e_{0}^{p}$	r
0.50	0.58	0.65	1

## Data Availability

The data presented in this study are available on request from the corresponding author.
